# Pediatric heart transplantation from donation after circulatory death using normothermic regional perfusion and cold storage from a distant donor: First US experience

**DOI:** 10.1016/j.xjtc.2023.05.010

**Published:** 2023-05-30

**Authors:** Reshma Biniwale, Saba Lahar, Shyamasundar Balasubramanya, Carla Caraccio, Biliet Ngang, Heather Barone, Emily Stimpson, Kim Dela Cruz, Juan Carlos Alejos, Ryan Williams, Nancy Halnon, Leigh Reardon, Ming-Sing Si, Richard Shemin, Abbas Ardehali, Glen Van Arsdell

**Affiliations:** a Division of Cardiothoracic Surgery, Department of Surgery, UCLA Health Sciences, Los Angeles, Calif; b Perfusion and ECMO Services, UCLA Cardiothoracic Surgery, Los Angeles, Calif; c Division of Pediatric Cardiology, Department of Pediatrics, UCLA Health Sciences, Los Angeles, Calif

**Figure:**
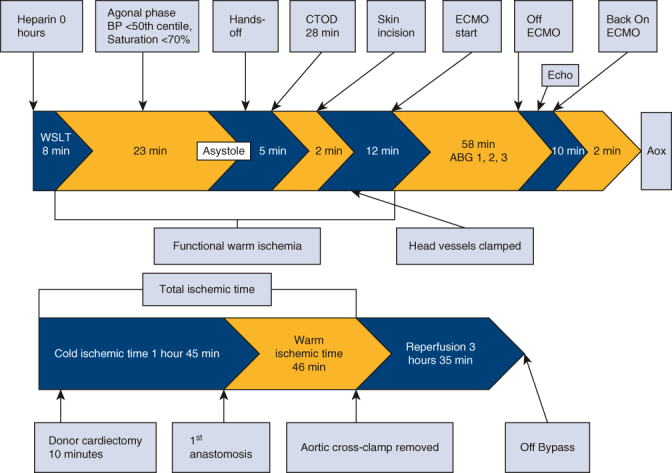
DCD NRP timeline.

**Central Message**

DCD NRP technique is a viable method to expand the pediatric heart donor pool.

The sole curative therapy for end-stage heart failure in pediatric patients is organ transplantation. Mechanical circulatory support in pediatrics is only a bridge to transplant or decision. In recent years, transplantation of hearts donated after determination of circulatory death (DCD) has been proposed as a novel method to increase the pool of donor allografts. In 2008, the first pediatric series of DCD implants used direct procurement and perfusion, followed by implantation in the recipient. In 2015, adult series of DCD donors with normothermic machine perfusion (NMP) from Australia as well as normothermic regional perfusion (NRP) from the United Kingdom with now 5-year follow-up^1^ were published. NMP is expensive, with the heart in a box machine exceeding $50,000 per patient.^2^ NMP does not allow for a functional assessment in a loaded state of the donor organ before transplantation. NMP technology is also not available for pediatric-sized donors. NRP is a thoracoabdominal in situ method of reperfusion of the transplantable organs using a modified extracorporeal membrane oxygenation (ECMO) circuit. NRP allows in situ organ assessment. The first pediatric DCD heart transplant using NRP occurred in 2019 in Belgium,^3^ and we report the first US DCD NRP experience in a 17-kg pediatric recipient.

IRB#21 to 001629 approval was obtained September 20, 2021. An 8-year-old 28-kg donor became available after being found unresponsive at home after a viral illness. The recipient was a 5-year-old redo heart transplant patient weighing 17 kg with left-sided pulmonary venous obstruction that had previously been stented. The recipient had decompensated due to coronary allograft vasculopathy requiring ECMO and Centrimag biventricular assist device complicated by a new stroke on computed tomography scan; however, the patient was responding to verbal stimuli and moving extremities. The recipient also was in renal failure on dialysis. Donor/recipient height ratio was 1.26 and weight ratio was 1.64. Informed consent for publication of data was obtained from the donor family for DCD donation as well as the recipient family. The donor was located 85 miles away from the accepting institution with a travel time of 20 minutes door to door via helicopter.

After systemic heparinization, the donor withdrawal of life support was performed by the donor hospital personnel per their hospital policy. The donor died within the acceptable time frame. After declaration of death and a 5-minute hands-off period, sternotomy was performed and the head vessels were clamped. Donor was expeditiously placed on NRP through the aorta and right atrial appendage. The NRP circuit was blood primed. A cardiac index approximately 2.0 L/min/m^2^ and age normal mean arterial pressure was maintained. This was sufficient flow to correct acidosis because cerebral blood flow was eliminated. The donor heart recovered in sinus rhythm within a minute of reperfusion, and was weaned off in an hour (Video 1) after correcting electrolyte and base deficits and transfusing packed red cells (Table 1). Abdominal organ dissection was completed during this time. Epicardiac echocardiography off NRP (Video 2) showed an ejection fraction of 50% with no valvular regurgitation. After returning to NRP briefly, the donor aorta was crossclamped and standard heart procurement carried out.

**Table 1:** Intraoperative donor functional assessment

Lab value	Preprocurement	First ABG NRP 0 min	Second ABG NRP 30 min	Third ABG NRP 45 min	Fifth ABG @ 60 min 5 min off NRP
pH	7.38	<7.0	7.29	7.51	7.53
Pco_2_	52	75	36	31	31
Po_2_	403	190	132	308	198
BE	–	–	−9	2	4
Bicarbonate	30	–	17.6	25.1	26.7
Sodium	160	149	154	155	157
Serum potassium on initiation of CPB (mmol/L)	3.2	5.9	3.1	3.7	3.5
Lactate on initiation of CPB (mmol/L)	na	12.58	13.69	13.48	11.68
Ionized calcium		0.9	0.98	1.01	1.1
Hematocrit (%)/hemoglobin		20/6.8	24/8.2	23/7.8	34/11.6
Echocardiogram LVEF (%)	63	na	na	55	60

*ABG*, Arterial blood gas; *NRP*, normothermic regional perfusion; *BE*, base excess; *CPB*, cardiopulmnary bypass; *na*, not available; *LVEF*, left ventricular ejection fraction.

Implantation of the heart required explantation of the Berlin cannulas and Centrimag biventricular assist device as well as sutureless repair of the left pulmonary veins. The aortic crossclamp was removed following the left atrial and aortic anastomoses. The heart was supported on bypass for additional time to complete the transplant, rewarm the patient, and support the right ventricle to avoid the need for postoperative ECMO.^1^ The heart returned in sinus rhythm with a 50% to 55% ejection fraction on epinephrine and milrinone drips. All times are summarized in Figure 1. The total ischemic time was 2 hours 32 minutes.

**Figure 1:**
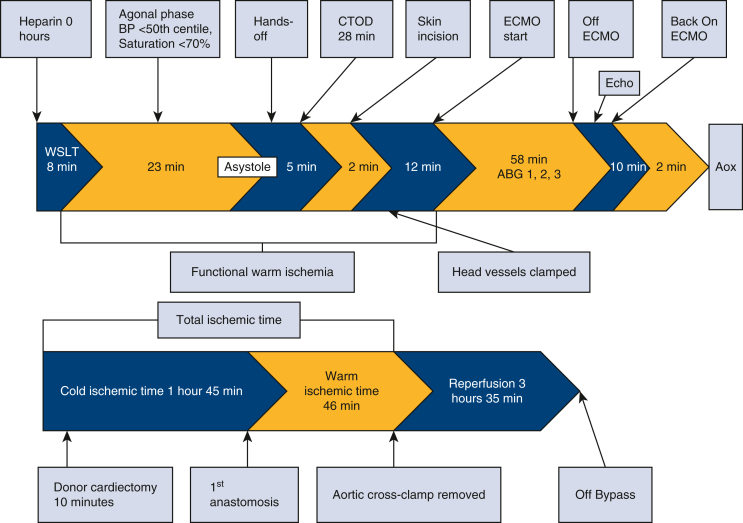
Donation after circulatory death normothermic regional perfusion timeline.

The recipient had a prolonged postoperative course to allow for renal recovery as well as physical rehabilitation and was discharged to home. Ejection fraction at discharge was 59%.

An analysis of the International Society for Heart and Lung Transplantation Registry to review the worldwide DCD pediatric experience demonstrated only (0.5%) of pediatric heart transplants were from DCD. There was no significant difference in the survival at 1 year between donor after brain death and DCD infant recipients.^4^ We hypothesize that DCD heart transplantation using NRP is a cost-effective technique that will safely expand the donor pool of available pediatric hearts and demonstrate similar outcomes to transplantation following donor after brain death.

## Supplementary Data

Video 1 Visual donor heart function at 60 minutes off normothermic regional perfusion. Video available at: https://www.jtcvs.org/article/S2666-2507(23)00184-0/fulltext.

Video 2 Epicardiac donor echocardiogram at 60 minutes off normothermic regional perfusion. Video available at: https://www.jtcvs.org/article/S2666-2507(23)00184-0/fulltext.
