# Supplementary figures and images for: Pediatric heart transplantation from donation after circulatory death using normothermic regional perfusion and cold storage from a distant donor: First US experience
Source: JTCVS Tech. 2023 May 30;20:158–61. doi: 10.1016/j.xjtc.2023.05.010 (PMC10405263; doi:10.1016/j.xjtc.2023.05.010)

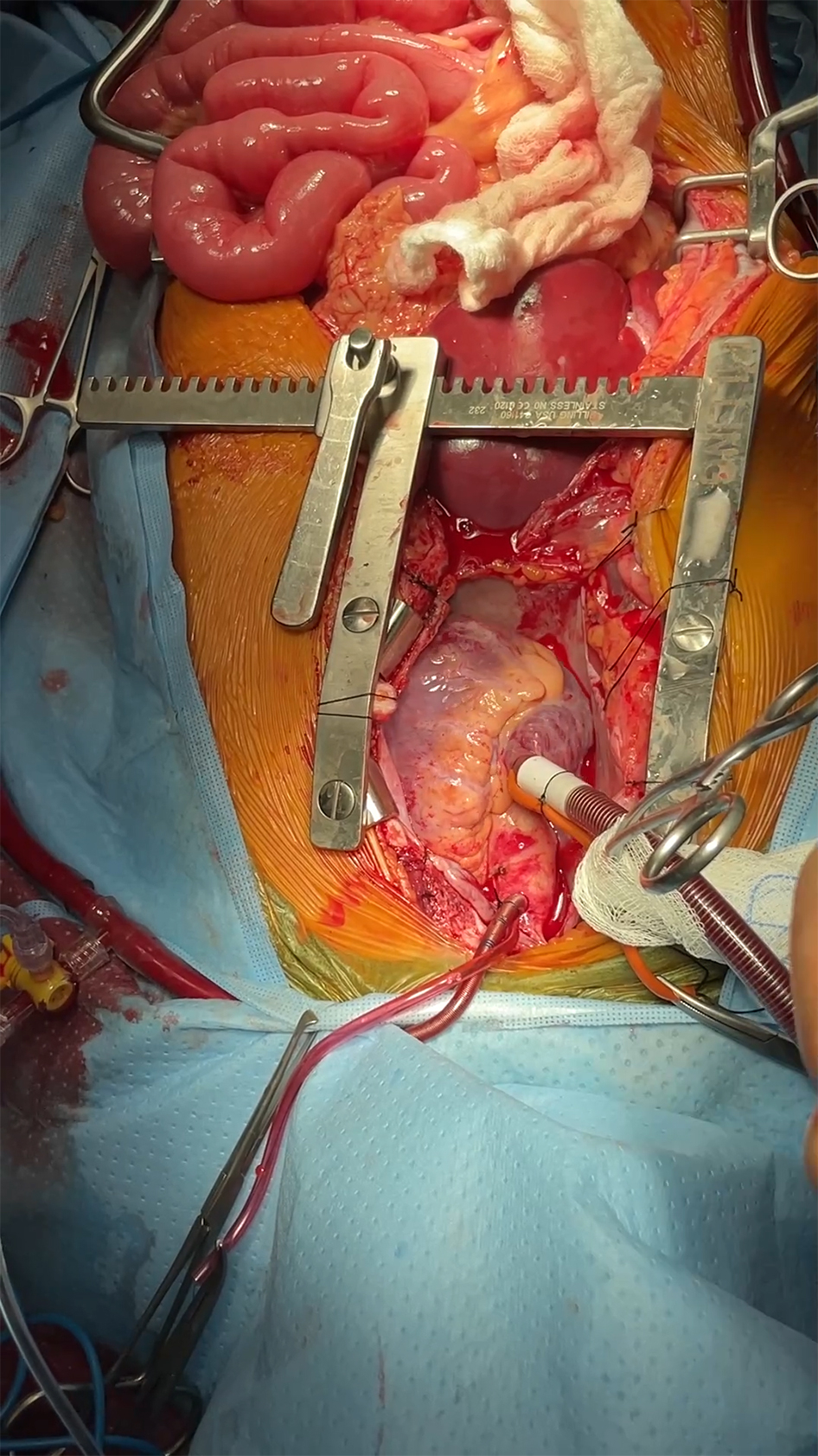

Supplement: Video 1 — Visual donor heart function at 60 minutes off normothermic regional perfusion. Video available at: https://www.jtcvs.org/article/S2666-2507(23)00184-0/fulltext. [file fx2.jpg]

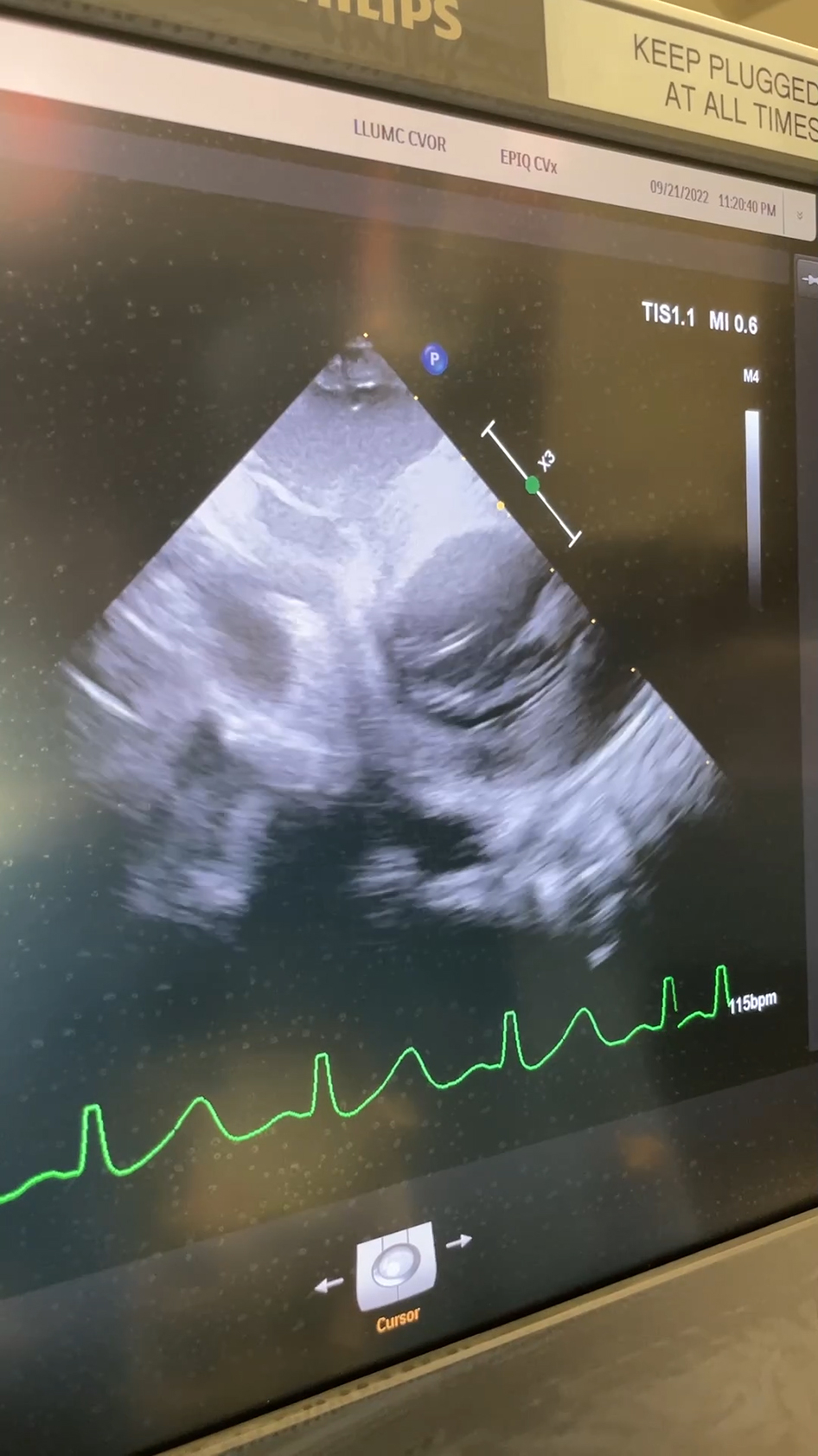

Supplement: Video 2 — Epicardiac donor echocardiogram at 60 minutes off normothermic regional perfusion. Video available at: https://www.jtcvs.org/article/S2666-2507(23)00184-0/fulltext. [file fx3.jpg]
